# The evolution of natural killer cell receptors

**DOI:** 10.1007/s00251-015-0869-7

**Published:** 2015-09-21

**Authors:** Paola Carrillo-Bustamante, Can Keşmir, Rob J. de Boer

**Affiliations:** 1 Theoretical Biology & Bioinformatics, Department of Biology, Utrecht University, Utrecht, The Netherlands; 2 Center for Modeling and Simulation in the Biosciences, Bio-Quant Center, Heidelberg University, Heidelberg, Germany

**Keywords:** NK cell receptors, Host-pathogen co-evolution, Agent-based modeling, KIR, Viral evasion

## Abstract

Natural killer (NK) cells are immune cells that play a crucial role against viral infections and tumors. To be tolerant against healthy tissue and simultaneously attack infected cells, the activity of NK cells is tightly regulated by a sophisticated array of germline-encoded activating and inhibiting receptors. The best characterized mechanism of NK cell activation is “missing self” detection, i.e., the recognition of virally infected or transformed cells that reduce their MHC expression to evade cytotoxic T cells. To monitor the expression of MHC-I on target cells, NK cells have monomorphic inhibitory receptors which interact with conserved MHC molecules. However, there are other NK cell receptors (NKRs) encoded by gene families showing a remarkable genetic diversity. Thus, NKR haplotypes contain several genes encoding for receptors with activating and inhibiting signaling, and that vary in gene content and allelic polymorphism. But if missing-self detection can be achieved by a monomorphic NKR system why have these polygenic and polymorphic receptors evolved? Here, we review the expansion of NKR receptor families in different mammal species, and we discuss several hypotheses that possibly underlie the diversification of the NK cell receptor complex, including the evolution of viral decoys, peptide sensitivity, and selective MHC-downregulation.

## Introduction

Natural killer (NK) cells are large granular cells that play a pivotal role in controlling viral infections and tumors (Vivier et al. 2008). To be tolerant to healthy tissue, and yet attack infected cells, the activity of NK cells must be tightly regulated. Unlike B and T cells, NK cells do not undergo gene rearrangements to generate the repertoire of cell surface receptors. Instead, they use germline-encoded inhibiting and activating receptors.

Inhibiting NK cell receptors is characterized by the presence of immunoreceptor tyrosine-based inhibitory motifs (ITIM) in their cytoplasmic tail that can decrease the state of activation (Vivier et al. 2004). Activating receptors lack ITIMs, but contain a positively charged amino acid (arginine or lysine) in their transmembrane region, and are associated with signaling adaptor molecules containing immunoreceptor tyrosine-based activating motifs (ITAM), such as DAP10, DAP12, or Fc *γ*R (Lanier 2005). NK cells integrate signals derived from both types of receptors upon cellular contact, thereby determining whether or not they should initiate effector functions.

Many inhibiting NK cell receptors interact with major histocompatibility complex (MHC) class I proteins, which are ubiquitously expressed on the surface of nucleated cells. Because of the abundant expression of MHC-I on many cells, NK cells remain non-responsive to healthy tissue. But when cells have a decreased expression of MHC-I, which can occur during certain viral infections or in tumors, they can become target for NK cell killing. The process by which NK cells detect cells with aberrant MHC-I expression has been coined by Kärre et al. as “missing-self” detection (Ljunggren and Kärre 1990).

For the development of functional NK cells in the bone marrow, interactions between inhibiting receptors and MHC-I are necessary (Raulet et al. 1997; Raulet and Vance 2006; Höglund and Brodin 2010). This process is called NK cell education and determines the threshold for activation in mature NK cells. Depending on the strength of the inhibitory signals received during development, every NK cell balances its activation threshold as a rheostat to adapt to the particular MHC phenotype of their host (Brodin et al. 2009). The expression of both activating and inhibiting receptors during development is thought to occur in a sequential and stochastic manner (Raulet et al. 1997; Moretta et al. 1990; Valiante et al. 1997; Husain et al. 2002), giving rise to a large NK cell repertoire composed of 3000–35,000 functionally different NK cell subsets (Horowitz et al. 2013).

## Evolution of NK cell receptors

**Fig. 1:**
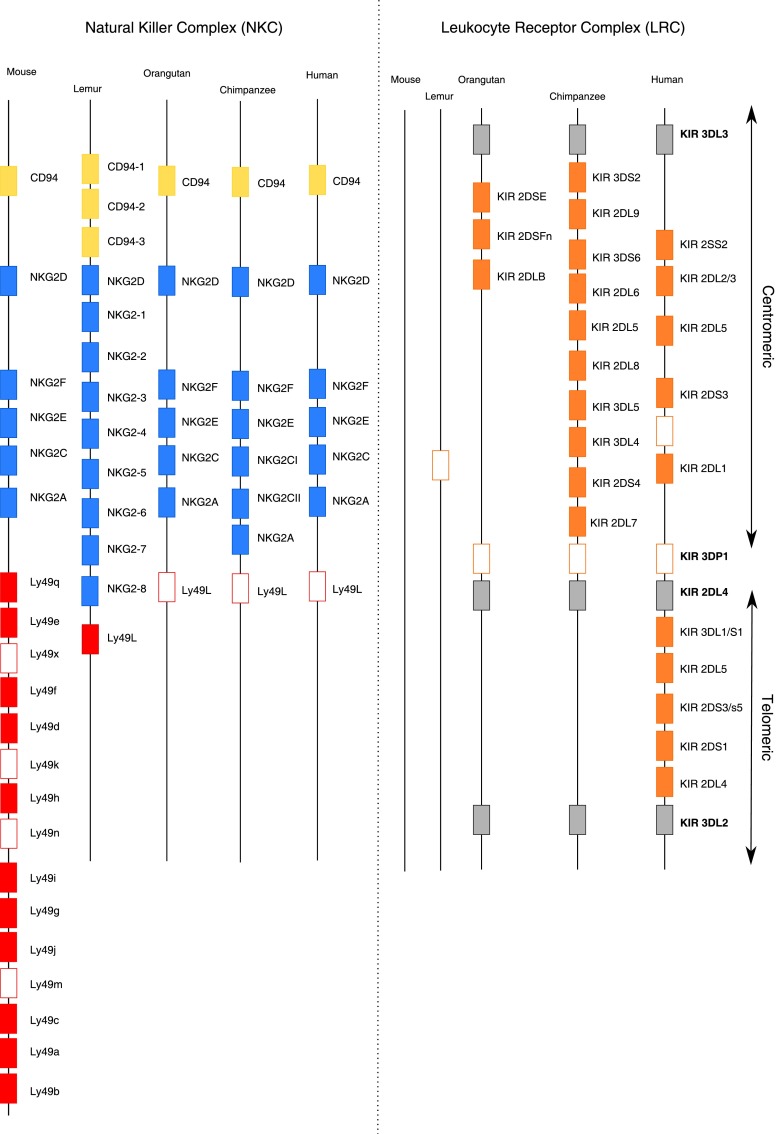
Cartoon of the NK cell receptor complexes. The NK cell receptor complexes. This figure shows a schematic organization of some of the genes encoded in the NKC (*left*) and in the KIR region of the LRC (*right*) for different species. The NKC encodes genes from the CD94 (*yellow boxes*), NKG2 (*blue*), and Ly49 (*red*) families. While higher primates have one copy of a non-functional Ly49 gene (*white boxes*), lemurs have one functional Ly49, and mice encode 15 Ly49 genes, 11 of which are functional. Lemurs have expanded their CD94/NKG2 system, with three CD94 genes, and eight NKG2 genes. KIRs (*orange boxes*) are encoded in the LRC. All higher primates share a common organization within this gene complex. Between the four framework genes, i.e. KIR3DL3, KIR2DL4, KIR3DL2, (*gray boxes*), and the pseudo gene KIR3DP1 (*white boxes*), the gene content varies across species. Lemurs have only one non-functional copy, and mice do not encode any KIR in the LRC. The gene order was taken from the literature sources mentioned in the text and from Kelley et al. (2005)

Genes encoding NK cell receptors are clustered in two main gene complexes: the natural killer complex (NKC) encoding C-type lectin-like molecules, and the leukocyte receptor complex (LRC), encoding the immunoglobulin-like receptors (Trowsdale 2001). Although these gene clusters are present in several species, there is extensive evidence for species-specific expansion of different NK cell receptor genes (Averdam et al. 2009; Futas and Horin 2013; Gagnier et al. 2003; Guethlein et al. 2007a, 2007b; Iizuka et al. 2003; Kelley et al. 2005; McQueen et al. 1998; Takahashi et al. 2004; Trowsdale 2001; Wilhelm et al. 2002, and see Fig. 1), resulting in a fascinating complexity of interactions between MHC-I and NK cell receptors. The NK cell receptor expansions known so far are described in detailed below.

### Expansion of NK cell receptors in primates

In humans, the main NK cell receptors for MHC-I are the killer immunoglobulin-like receptors (KIRs), which are located in the LRC on chromosome 19q13.4 (Wende et al. 1999). The haplotypes encoding KIRs exhibit great differences in gene content and allelic polymorphism, with up to 17 genes encoded over approximately 150 kilo bases (Parham 2005; Martin et al. 2000; Wende et al. 1999; Barten et al. 2001; Wilson et al. 2000; Trowsdale 2001). The marked differences in gene content are thought to be the result of non-reciprocal crossovers in the tandemly arranged genes, causing hybrid loci or contraction and expansion of the haplotype (Vilches and Parham 2002; Martin et al. 2003; Martin et al. 2000; Wilson et al. 2000; Wende et al. 1999).

KIRs can have either two (KIR2D) or three (KIR3D) extracellular immunoglobulin-like domains and contain either long cytoplasmic tails with ITIM motifs or short cytoplasmic tails comprising ITAMS (Vilches and Parham 2002). An exception is KIR2DL4 which has a cytoplasmic long tail and possesses a positively charged residue in the transmembrane region, allowing association with the activating protein Fc *γ*R (Kikuchi-Maki et al. 2005).

KIRs started expanding between 31 and 40 million years ago (Martin et al. 2000), resulting in a rapid and species-specific diversification in primates (Martin et al. 2000). Old world monkeys, apes, and humans have a common organization of the KIR gene complex, sharing four phylogenetic lineages (I,II,III, and V), which are characterized by their structure and specificity for MHC-I (Parham and Moffett, 2002; Vilches and Parham 2013 and see Table 1). In humans, the lineage I KIR binds HLA-G-peptide complexes (Rajagopalan 2010), lineage II KIRs recognize epitopes A3/A11 on HLA-A, and Bw4 on HLA-A and B, and lineage III KIRs bind to HLA-C epitopes C1 and C2 (Trowsdale et al. 2001). These epitopes are mutually exclusive and differ in unique residues that are involved in the KIR-pMHC interaction (Trowsdale et al. 2001). The dimorphism among HLA-C molecules at position 80, i.e., either asparagine or lysine, determines the classification of HLA-C1 and HLA-C2 alleles (Colonna et al. 1993; Fan et al. 2001). The Bw4 epitope, on the other hand, is defined by residues 77–83 (Sanjanwala et al. 2008; Gumperz et al. 1997; Gumperz et al. 1995). As shown by a vast range of structural and functional studies (Colonna et al. 1993; Vivian et al. 2011; Schafer et al. 2014; Saunders et al. 2015), single amino acid substitutions in these key residues can have large effects on KIR binding. Ligands for KIR lineage V have to still be identified.

**Table 1:** Ligands of activating and inhibiting human KIRs

	Lineage	Ligand
Activating KIR		
2DS1	III	HLA-C2
2DS2	III	HLA-C1, HLA-A*11:01
2DS3	III	unknown
2DS4	III	HLA-C*05:01, A*11:02, C*16:01
2DS5	III	unknown
3DS1	II	unknown
Inhibiting KIR		
2DL1	III	HLA-C2
2DL2 / 2DL3	III	HLA-C1, HLA-C2, HLA-B*46:01, and
		HLA-B*73:01 (C1 epitope)
2DL4	I	HLA-G (might be an intracellular
		interaction)
2DL5	I	unknown
3DL1	II	HLA-A with Bw4 motif, HLA-Bw4
3DL2	II	HLA-A3/A11
3DL3	V	unknown

In the LRC, there are some differences in gene content and specificity of the KIRs across primate species. Importantly, the marked differences go hand in hand with the evolution of MHC-I genes. In macaques, carrying duplicated MHC-A and MHC-B genes (Daza-Vamenta et al. 2004; Shiina et al. 2006), the Bw4 motif is important for binding of a large collection of several lineage II KIRs (Adams and Parham 2001; Bimber et al. 2008; Blokhuis et al. 2010, 2011; Kruse et al. 2010; Schafer et al. 2014). Consistent with the observation that macaques lack MHC-C molecules, they have only one lineage III KIR, with members that do not seem to bind any MHC-class I (Bimber et al. 2008; Hershberger et al. 2001). Orangutans, on the other hand, carry fewer MHC-A and MHC-B loci than macaques, and encode only one lineage II KIR accordingly. Orangutans were the first primates to evolve MHC-C (C1 epitope), corresponding to the expansion of their lineage III KIRs (Guethlein et al. 2007b). Lineage III KIRs expanded further in chimpanzees, correlating with the evolution of the C2 epitope in MHC-C. Chimpanzees have both inhibiting and activating KIRs, and eight of them recognize MHC-C only (Abi-Rached et al. 2010).

Humans, in contrast, have only seven lineage III KIRs and two lineage II KIRs, with three KIR genes showing specificity for HLA-C, including the inhibiting KIR2DL2/3, and KIR2DL1, and the activating KIR2DS2 (see Table 1). Additionally, humans are the only species that have undergone specific expansion in the telomeric part of the KIR complex (Abi-Rached et al. 2010). While the centromeric part of human KIR haplotypes is more similar to chimpanzee KIR haplotypes (Abi-Rached et al. 2010), the telomeric region in humans accumulated genes that show mainly activating potential, and that have little or no binding affinity to HLA-I molecules, such as KIR2DS2, 2DS3, and 2DS5 (Moesta et al. 2010; Pyo et al. 2010). This clear distinction between centromeric and telomeric genes allowed for the distinction of the two haplotype groups, A and B. Both haplotypes are present in all human populations (Hollenbach et al. 2010) (including Japanese Yawata et al. 2006; Amerindian Gendzekhadze et al. 2009; African Norman et al. 2013; Polynesian Nemat-Gorgani et al. 2014), differ in frequency and are maintained by balancing selection (Yawata et al. 2006), indicating their essential role for long-term survival (Gendzekhadze et al. 2009).

The other receptor cluster in primates is the NKC. The main members of these gene families are the Ly49 and the NKG2 genes. Primates have only one gene of the Ly49 family, which is a pseudo gene, but their NKC encodes several NKG2 genes (Renedo et al. 1997; Khakoo et al. 2000; LaBonte et al. 2001; Guethlein et al. 2002). Members of the NKG2 family include the inhibiting NKG2A, the activating NKG2C, NKG2E, and NKG2D, and the NKG2F, for which no function has been yet determined (Lazetic et al. 1996). NKG2 proteins dimerize with the invariant CD94 molecule on the cell surface, which contains a short cytoplasmic domain and transduces the activating or inhibiting signal (Lazetic et al. 1996). An exception is NKG2D, an activating receptor, which shares little sequence similarity with the other members, and associates with the activating molecule DAP10 on the cell surface.

The ligands of NKG2A and NKG2C include the conserved and non-classical HLA-E molecule in humans and Qa-1 ^*b*^ in mice (Borrego et al. 1998; Braud et al. 1998; Petrie et al. 2008; Zeng et al. 2012), which present peptides derived from the leader sequences of the classical HLA-A, HLA-B, and HLA-C molecules in humans, and from H2 molecules in mice. The engagement of NKG2A by HLA-E or Qa-1 ^*b*^ inhibits the activity of NK cells, preventing target cell lysis. In higher primates, both NKG2A and MHC-E (i.e., receptor and ligand) are very well conserved (Shum et al. 2002), presenting a system for detection of “gross” MHC-I expression, that unlike KIRs is highly conserved.

Lemurs, on the other hand, exhibit only one single non-functional KIR gene in their LRC, but they have diversified the genes encoding CD94 and NKG2 (Averdam et al. 2009). Located in lemur chromosome 7, the NKC comprises three CD94 genes and five to eight inhibiting and activating genes. Like KIRs in higher primates, the CD94 and NKG2 genes in lemurs are highly polymorphic, with many of the polymorphic positions representing functionally relevant sites, i.e., residues involved in binding of MHC class I ligands and their presented peptides (Averdam et al. 2009). The homologs of HLA-E have not been yet identified in prosimians, but the ligands for the NKG2 receptors are expected to be the lemur MHC-I molecules (Averdam et al. 2009).

Importantly, Averdam et al. showed that all possible CD94/NKG2 combinations are able to form heterodimers at the cell surface, giving rise to a great combinatorial diversity. For instance, the combination of three CD94 and five NKG2 molecules in the gray mouse lemur or three CD94 and eight NKG2 molecules in the ruffed lemur gives rise to 15 or 24 different NK cell receptors, respectively, (Walter 2011; Averdam et al. 2009). An exchange of the CD94 or the NKG2 subunit can influence the binding specificity for MHC class I ligands, changing the functionality of the NK cell receptors (Averdam et al. 2009). Thus, lower primates seem to have evolved an alternative system for variable NK cell receptors.

### Expansion of NK cell receptors in rodents

The LRC is located on chromosome 7 in mice and on chromosome 4 in rats (Kirkham and Carlyle 2014; Iizuka et al. 2003; Schenkel et al. 2013). The murine LRC does not contain any of the KIRs that bind MHC-I in humans (Martin et al. 2002a), but contains orthologs of human GP6 (Trowsdale et al. 2001), NCR1, RPS9, and LAIR1 (Martin et al. 2003), and genes of the Pir family, which share sequence identity with the human LILRs (Kubagawa et al. 1999).

Instead of having polygenic and polymorphic KIRs, rodents have expanded their Ly49 genes, resulting in a remarkable diversity across different inbred mouse strains (Kirkham and Carlyle 2014; Iizuka et al. 2003). While the mouse Ly49 complex comprises at least 20 genes and pseudo genes (Wilhelm et al. 2002), the variation is even larger in rats, with 19 functional genes and 15 pseudo genes (Nylenna et al. 2005; Flornes et al. 2010). Table 2 shows the most important known receptors in mouse strains studied so far (Rahim et al. 2014). Ly49 receptors in mice are functionally similar to KIRs in humans, having both inhibiting and activating receptors, and genes encoding proteins that preferentially bind mouse MHC-I (Schenkel et al. 2013). Although several ligands for activating Ly49 receptors remain unknown, some activating receptors bind viral encoded proteins (see below).

**Table 2:** Ly49 haplotypes in four known mouse strains with their response to MCMV (modified from Rahim et al. 2014)

Mouse strain			
NOD	129	B6	BALB
Response to MCMV			
Susceptible	Susceptible	Resistant	Susceptible
Activating			
Ly49D	Ly49P	Ly49D	Ly49L
Ly49H	Ly49R	Ly49H	
Ly49M	Ly4UP		
Ly49P _1_			
Ly49P _3_			
Ly49U			
Ly49W			
Inhibiting			
Ly49A	Ly49B ^*b*^	Ly49A	Ly49A
Ly49B ^*b*^	Ly49E	Ly49B ^*b*^	Ly49B ^*b*^
Ly49C	Ly49EC _2_	Ly49C	Ly49C
Ly49E	Ly49G ^*b*^	Ly49E	Ly49E
Ly49F	Ly49I$_{1} ^{b}$	Ly49F	Ly49G
Ly49G _2_	Ly49O	Ly49G	Ly49I
Ly49I	Ly49Q _1_	Ly49I	Ly49Q
Ly49Q	Ly49S	Ly49J	
	Ly49T	Ly49Q	
	Ly49V		

### NK cell receptors in other species

At least five highly conserved polymorphic Ly49 genes have been found in some equids, including horses, asses and zebras (Takahashi et al. 2004; Futas and Horin 2013). By contrast, only one single Ly49 has been found in cattle (McQueen et al. 1998), domesticated dogs and cats, and pigs (Gagnier et al. 2003). Cattle have also functional KIRs (Parham and Moffett 2013; Guethlein et al. 2007a; Allan et al. 2015). Opposite to primate KIRs, which diverged from the founder gene KIR3DL, cattle expanded their founder gene KIR3DLX. In every species, the gene that was not diversified became nonfunctional (Guethlein et al. 2007a; Dobromylskyj and Ellis 2007). No species studied so far is known to have two expanded NK cell receptor families (Parham and Moffett 2013), but several species diversify neither, keeping both KIR and Ly49 as one single copy genes (Hammond et al. 2009).

## Why are NK cell receptors polygenic and polymorphic?

The evolution of variable NK cell receptor genes is expected to have been shaped by several factors determining fitness and survival, like pathogen resistance, detection of polymorphic ligands like MHC-I, and reproductive success (Parham and Moffett 2013). The inter- and intra-species gene diversity indicates their rapid evolution. Importantly, the independent convergent evolution of variable NK cell receptors in several different species highlights their functional importance. However, the exact evolutionary selection pressure whereby NK cell receptors became polymorphic and polygenic remains unresolved. The conserved inhibitory receptor NKG2A demonstrates that abnormalities in MHC-I expression, i.e., missing-self, can be detected without a polygenic and polymorphic NK cell receptor system. Why then have these polygenic and polymorphic receptors evolved?

### Reproductive success

Since the divergence from chimpanzees, hominids have evolved several changes in aspects of locomotion, anatomy, and reproduction. Two key aspects of this human specific evolution affect the reproductive success: bipedalism and larger brain size. The evolution of bipedalism imposed drastic anatomic changes in the size and shape of the human female pelvis, affecting directly the size of the birth canal. While the size of the birth canal was decreasing, the evolution of larger brains was imposing additional challenges for a successful birth (Parham and Moffett 2013).

The evolution of bigger brain sizes required more blood supply in the placenta (Leonard et al. 2007), a process that has been achieved by a remodeling of the uterine arteries (reviewed in Wallace et al. 2012). Arterial remodeling occurs thanks to extra-villous trophoblast (EVT) cells. EVT are fetal cells invading the uterus, transforming the spiral arteries into large vessels that are able to provide adequate blood supply to the growing fetus (Moffett-King 2002). Preeclampsia and recurrent miscarriage have been associated with a compromised arterial remodeling (Wallace et al. 2012).

A successful EVT invasion depends on the interactions of EVT with uterine NK cells. The activation of uterine NK cells is important for arterial remodeling, as it results in the release of cytokines, which in turn promote migration of the trophoblasts (Xiong et al. 2013). Because EVT uniquely express HLA-C (lacking HLA-A or HLA-B), and uterine NK cells preferentially express HLA-C-specific KIRs (Sharkey et al. 2008), several correlations between reproductive success and particular KIR/HLA-C combinations have been found (Hiby et al. 2004, 2010, 2014). The presence of HLA alleles in the fetus binding more inhibiting than activating receptors of the mother’s NK cells results in compromised arterial remodeling and reduced fetal growth (Kieckbusch et al. 2014). Accordingly, mothers being homozygous for KIR A haplotypes have a high risk of developing preeclampsia if the fetus carries one C2 allele, as KIR A homozygous individuals have two copies of the inhibiting KIR2DL2, which binds strongly to C2 (Hiby et al. 2004, 2010, 2014).

The evolution of larger brain sizes started mainly in *Homo erectus* (Robson and Wood 2008) and correlates with the emergence of KIR B haplotypes that encode more activating KIRs than KIR A haplotypes. However, the evolution of more activating KIRs is not always beneficial for reproduction, as NK cell-mediated placentation can lead to large babies that are not able to pass through the birth canal, causing obstructed labor (Hiby et al. 2014). A successful placentation is hence dependent on a tight NK cell-mediated regulation. Thus, the pressure for a successful reproduction could drive and maintain inhibiting and activating receptors specific for MHC-I. However, because humans are the only species with a narrow birth canal requiring deep placentation (Moffett and Loke 2006), it is not likely that reproductive success would exert sufficient selection pressure to expand and maintain a set of polygenic and polymorphic NK cell receptors in other species.

### Response to viral infections

Because of the evolutionary arms race between infectious agents and the host’s immune system, another possible explanation for the diversification of NK cell receptors is the selection pressures imposed by various successful immuno-evasive mechanisms evolved by several pathogens (Lanier 2008; Sun and Lanier 2009). There is extensive evidence of associations between particular NKRs and the viral control caused by viruses, including cytomegalovirus (CMV), human immunodeficiency virus (HIV-1), and hepatitis C virus (HCV).

#### Role of KIRs in human diseases

Several human studies have provided evidence that some NKRs may be directly involved in viral control. Associations between particular KIR alleles and disease outcome have been found in HIV-1, HCV, and Influenza (Jamil and Khakoo 2011).

During HIV-1 infection, there is an expansion of 3DS1 ^+^NK cells, and this expansion is dependent on the presence of the Bw4-80I epitope (Alter et al. 2009; Pelak et al. 2011). The expansion of selected NK cell subsets could be beneficial to the host due to an immediate and strong NK cell response. Indeed, individuals carrying these KIR-HLA combinations showed lower viremia and a slower progression to AIDS (Martin et al. 2002b). Furthermore, an increased number of KIR3DS1 (caused by a higher copy number variants of KIR3DL1/S1) was correlated with a lower set viral point in the presence of HLA-Bw4-80I (Pelak et al. 2011). All these studies show an important role of the KIR3DS1- Bw4-80I pair in the immune response to HIV infection. However, there is no evidence for direct binding between KIR3DS1 and HLA-Bw4 molecules (Gillespie et al. 2007; O’Connor et al. 2007; Carr et al. 2007), suggesting that the KIR3DS1 ligand interactions might be finely regulated, e.g., via the presented peptide (O’Connor et al. 2015).

NK cells expressing the inhibiting receptors 2DL2/L3 exhibit increased degranulation when they respond to HCV infected target cells (Amadei et al. 2010). Both 2DL3 and 2DL2 bind HLA-C1 epitopes, but they differ in their binding affinities, with 2DL2 binding stronger than 2DL3 (Winter et al. 1998). Because of the weaker inhibiting interaction, homozygous individuals for the 2DL3-HLA-C1 pair control HCV infection better and can even experience spontaneous clearance (Khakoo et al. 2004; Romero et al. 2008). Additionally, a protective effect of the activating 3DS1 in combination with HLA-Bw4-I80 in hepatocarcinoma has been found in patients with chronic HCV infection (López-Vázquez et al. 2005).

Studies of human influenza A virus (IAV) have also shed light on NK cell responsiveness depending on KIR-HLA genotypes. As shown by multicolor flow cytometry, NK cells from individuals homozygous for the 2DL3-C1 pair had a stronger activation (i.e., IFN- *γ* secretion and degranulation) to IAV infected cells than those homozygous for 2DL1-C2 (Ahlenstiel et al. 2008). Another study of patients infected with 2009 pandemic IAV strain (H1N1/09) established that patients suffering from severe and pathological reactions against the virus lack functional receptor-ligand pairs (La et al. 2011). Patients carrying inhibiting 3DL1 but lacking HLA-Bw4 molecules, as well as patients positive for 2DL1 and lacking HLA-C2 alleles, were higher in ICU patients relative to healthy controls or patients with mild reactions against H1N1/09. This study suggests that a lack of KIR-mediated inhibition might lead to NK cell dysfunction and with it to an immunopathological outcome.

These few examples mirror the large range of studies associating KIR-MHC combinations with the outcome of diseases. However, the lack of well-characterized ligands for several receptors and specific monoclonal antibodies for detecting specific KIRs limit the understanding of the precise molecular mechanisms underlying this associations and with it the precise functional role of these receptors upon infection.

#### Selective downregulation of MHC-I

Several viruses, including Epstein-Barr-Virus (EBV), CMV, and HIV decrease the expression of MHC-I on the cells they have infected to escape from the T cell immune responses. Interestingly, the downregulation does not always affect all MHC molecules in the same way, and some viruses, e.g., HIV and CMV, have evolved mechanisms that target only particular loci (reviewed in Nash et al. 2014). In humans, the HLA molecules presenting peptides to T cells (A and B loci) tend to be downregulated, while those HLA molecules inhibiting NK cells (C and E loci) remain unchanged, suggesting that selective MHC downregulation could be a viral strategy to avoid missing-self detection (Table 3).

**Table 3:** Viral proteins inducing locus-specific MHC-I downregulation in humans

Virus protein	Downregulated HLA allotypes	Expressed HLA allotypes
HCMV US2/US11	HLA-A, HLA-B	HLA-C, HLA-E, HLA-G
HCMV UL40	not applicable	HLA-E
HIV Nef	HLA-A, HLA-B	HLA-C
KSHV K5	HLA-A, HLA-B, HLA-C (weakly)	HLA-E
EBV BILBF 1	HLA-A, HLA-B, HLA-E	HLA-C

HCMV encodes several immunoevasin proteins that selectively downregulate the expression of MHC-I on the cell surface (Nash et al. 2014), such as US2 and US11, targeting specific and non overlapping HLA-A and HLA-B molecules, by promoting their export into the cytosol for proteosomal degradation (Llano et al. 2003; Gewurz et al. 2001; Schust et al. 1998). In addition to selective MHC-downregulation, HCMV encodes proteins that enhance MHC-I expression to inhibit NK cells. For example, the peptides from UL40 have a high sequence similarity to peptides from HLA-C alleles (Tomasec et al. 2000; Ulbrecht et al. 2000). By binding to HLA-E, UL40 peptides can promote its expression on the cell surface, providing a ligand for NKG2A.

HIV-1 also decreases the expression of particular HLA alleles. HIV Nef binds to the cytoplasmic tails of the HLA-A and HLA-B molecules in the ER, re-directing them to endolysosomal compartments for degradation (Schaefer et al. 2008). Small differences in the cytoplasmic tails of HLA-C and HLA-E prevent Nef from hampering their transport to the cell surface, which in turn prevents HIV-infected cells to be lysed by most NK cells (Gall et al. 1998; Collins et al. 1998; Cohen et al. 1999).

Kaposi’s sarcoma-associated herpesvirus (KSHV) is another example of viruses that can evade CTL responses via locus-specific MHC-I downregulation. KSVH encodes two membrane-bound ubiquitin E3 ligases, K3 and K5, which induce rapid internalization and degradation of MHC-I molecules (Coscoy and Ganem 2000; Ishido et al. 2000), yet with different specificities (Ishido et al. 2000). While K3 downregulates all four HLA allotypes (i.e., HLA-A, HLA-B, HLA-C, and HLA-E), K5 impairs the expression of HLA-A and HLA-B, weakly downregulates HLA-C, but does not affect the expression of HLA-E (Ishido et al. 2000). Similarily, the EBV encoded protein BILF1 induces the rapid degradations of multiple HLA-A, -B, and -E molecules, but hardly affects the expression of HLA-C (Griffin et al. 2013).

Mouse CMV (MCMV) encodes glycoproteins that interfere with the expression of MHC-I molecules (Wagner et al. 2002). For example, gp40 retains the MHC molecules in the ER (Ziegler et al. 1997), while gp48 re-routes mature MHC to endo-lysosomal compartments for degradation (Reusch et al. 1999). Balancing this broad MHC-I downregulation is the MCMV protein gp34, which escorts some MHC alleles to the cell surface (Kleijnen et al. 1997), and thereby allows infected cells to escape NK cell mediated killing.

All these examples highlight the evolutionary importance of viruses partially downregulating the expression of MHC-I molecules. The specific targets for MHC downregulation (such as in HCMV or in HIV-1) illustrate the adaptation of the viruses to several MHC-I loci. By selectively downregulating MHC-I molecules, some viruses escape from NK cell responses. Thus, it is possible that the evolution of these various immunoevasins was driven by the selection pressures imposed by inhibiting NK cell receptors.

Our in silico studies of evolving host populations infected with herpes-like viruses that selectively downregulate one of the two MHC loci in the host (Carrillo-Bustamante et al. 2015b) showed that selective MHC downregulation exerts selection pressure to evolve specific NK cell receptors. Due to the infection with such viruses hosts naturally evolve inhibiting receptors that specialize to one MHC locus, while loosing their binding affinity to most MHC molecules in the other locus (Carrillo-Bustamante et al. 2015b). Importantly, the evolution of these “MHC locus” detectors exploits the similarity of the MHC alleles within each locus and depends on the difference between MHC loci. The easier it is to classify an MHC allele to its locus, the easier it is to evolve “locus specific” detectors, decreasing the selection pressure to diversify the genes encoding NK cell receptors. However, if MHC molecules in one locus resemble alleles from another locus, it becomes difficult for inhibiting receptors to discriminate between different MHC-I loci, driving the evolution of highly specific inhibiting receptors encoded in polygenic haplotypes.

These studies confirm the importance of MHC molecules on the evolution of NK cell receptors and are in line with the observation that inhibiting KIRs recognize motifs shared by common MHC epitopes, such as HLA-A3/A11, HLA-Bw4, HLA-C1, and HLA-C2 (Trowsdale et al. 2001). However, only the HLA-C ligands are locus specific, as the Bw4 epitope is also carried by 25 % of HLA-A alleles (Gonzalez-Galarza et al. 2011), impeding the discrimination between HLA-A and HLA-B. Moreover, most HLA-A and HLA-B alleles (approximately two thirds in each locus) do not carry these KIR-specific epitopes (Gonzalez-Galarza et al. 2011), indicating that humans have not evolved optimal HLA-A or HLA-B detectors. Nevertheless, our results are in perfect agreement with the emergence of MHC-C specific detectors in chimpanzees and humans (i.e., lineage III KIRs). Why only MHC-C specific detectors have evolved remains puzzling and suggests that additional evasion mechanisms must have been involved in the evolution of MHC-A and MHC-B specific KIRs.

#### MHC-I decoys in CMV

In addition to selectively downregulating the expression of MHC I molecules, some viruses use MHC-I like proteins, i.e., decoys, that can directly interact with the NK cell receptors to modulate the immune response. Examples of such decoys are the HCMV encoded UL18 binding to the inhibitory LIR-1 (Prod’homme et al. 2007), and the MCMV encoded m144, mimicking key structural characteristics of H-2 molecules (Prod’homme et al. 2007; Natarajan et al. 2006). Interestingly, UL18 and m144 share more sequence similarity with MHC-I than they share with each other, showing that species-specific immune pressure led to independent acquisition of MHC-I mimics (Natarajan et al. 2006; Farrell et al. 1997).

The most extensively studied MHC decoy is the MCMV encoded m157 protein. m157 allows MCMV to avoid NK cell activation by engaging inhibitory receptors with high affinity, as shown in 129/J mice that are highly susceptible to MCMV infection (Smith et al. 2002). Unlike 129/J mice, C57BL/6 mice exhibit spontaneous resistance against MCMV, a phenomenon that has been genetically mapped to one single gene encoding the activating receptor Ly49H (Lee et al. 2001; Brown et al. 2001), which also binds m157 with high affinity (Lee et al. 2001; Smith et al. 2002). The activating Ly49H evolved from its inhibitory counterpart Ly49I (Abi-Rached and Parham 2005), indicating that the evolution of the activating receptors is a result of the novel selective pressure exerted by CMV after evolving MHC-I decoys (Arase and Lanier 2002; Sun and Lanier 2009; Lanier 2008). The immuno-evasive role of m157 is further supported by studies of wild mouse-derived MCMV isolates where several strong interactions between m157 and an array of inhibitory receptors were detected. Only a few m157 variants engage the activating Ly49H receptor (Corbett et al. 2011; Voigt et al. 2003), indicating that the host’s protection mediated by Ly49H is rather uncommon among wild mouse populations. Importantly, NK cell responses also exert strong immune pressure on the virus, as shown by experiments where the repeated passage of MCMV through resistant Ly49H mice resulted in the rapid evolution of m157 mutants that do not engage Ly49H, and thereby escape from the NK cell immune response (Voigt et al. 2003).

C57BL/6 is not the only inbred strain resistant to MCMV. Inbred MA/My mice also have low viral titers after infection with MCMV, although they do not possess the Ly49H gene. Their resistance is mediated by the activating receptor Ly49P which specifically recognizes MCMV infected cells in a H2-D ^k^ dependent manner (Kielczewska et al. 2009). Interestingly, this resistance requires the presence of the virally encoded protein m04, which escorts and binds newly assembled MHC-I molecules on the cell surface. Other activating receptors that recognize MCMV infection in a m04-H2 dependent manner include Ly49L ^BALB^, Ly49P1 ^NOD^, and Ly49W1 ^NOD^ (Pyzik et al. 2011). Like m157, the original function of m04 might have been to counteract the effect of MHC downregulation and avoid “missing-self” detection by inhibiting receptors. Together, these observations suggest that hosts evolved novel activating receptors to recognize the decoys evolved by viruses.

With a computational agent-based model of co-evolving hosts and CMV-like viruses (Carrillo-Bustamante et al. 2013, 2014, 2015c), we tested the effects of MHC-I decoys on the evolution of NK cell receptors. Our computational approach revealed that viruses evolving MHC-decoys drive the evolution of highly specific inhibiting receptors, i.e., receptors that specialize to particular MHC molecules in the population. Because inhibiting NK cell receptors face the challenge to avoid being “fooled” by the viral molecules mimicking MHC-I, such a high degree of specificity is optimal during infections with viruses evolving decoys. Similarly, activating receptors are beneficial in our in silico populations because of the protection they provide when they bind MHC-I decoys, thereby activating NK cells. Given their involvement in pathogen defense and host survival, we observe a natural expansion of the NK cell receptor cluster, evolving several haplotypes composed of specific inhibiting and activating genes.

Although these computational models provide insightful mechanistic insights into this evolutionary model, several questions remain still open. If activating NK cell receptors are indeed advantageous because they can recognize viral products, why is it so challenging to find and characterize ligands for them? It is possible that these viral ligands are encoded by short undetermined sequences in viruses having large genomes, such as CMV, EBV, and other viruses from the Herpes family. Another possible explanation for the lack of identified viral ligands is that viruses evolve rapidly, remaining a moving target for activating receptors, hence to impede their adaptation.

#### Peptide sensitivity

It is widely believed that, unlike T cell receptors, inhibiting KIRs (iKIRs) is not very specific for particular peptide-MHC (pMHC) complexes. However, several studies have shown that iKIRs can be sensitive to the specific peptides bound by the HLA molecules (Malnati et al. 1995; Rajagopalan and Long 1997; Peruzzi et al. 1996; Thananchai et al. 2007; Hansasuta et al. 2004). Crystal structures of KIR2DL1 and KIR2DL2 in complex with their HLA-C ligands further supported this observation (Boyington et al. 2000; Brooks et al. 2000; Fan et al. 2001; Li and Mariuzza 2014), by revealing that specifically positions 7 (P7) and 8 (P8) of the bound peptide are in direct contact with residues of the iKIR. Other studies showed that peptides can markedly reduce or increase KIR-mediated inhibition (Cassidy et al. 2014; Fadda et al. 2010).

Because of the direct contact between iKIR and the MHC presented peptides, NK cell activation may vary in a peptide-dependent manner, making iKIRs sensitive to changes in the peptide repertoire presented by MHC-I molecules. These observations call for an extension of the current model of NK cell activation: “missing self” detection could be extended by “altered self”, where changes in the MHC-I peptide repertoire modulate NK cell signaling.

The importance of peptide sensitivity has been recently emphasized by HIV-1 studies, demonstrating that sequence variations within HLA-C restricted HIV epitopes disrupt or promote the binding to inhibiting KIR2DL2, subsequently modifying NK cell activation (Fadda et al. 2012; van Teijlingen et al. 2014). Importantly, these studies show that a small number of naturally occurring variants of HIV-1 epitopes that are presented by HLA-C*03:04 can strongly engage KIR2DL2, inducing a strong inhibiting signal for the NK cells (van Teijlingen et al. 2014). It is tempting to speculate that viral variants are selected to avoid NK cell mediated immune responses in individuals expressing the corresponding KIR/HLA pair.

The changes in the MHC presented peptides after a viral infection are also expected to enhance the binding to activating receptors, allowing for NK cell activation. In mice, the interactions between the activating CD94/NKG2E and the peptides loaded onto the non-classical MHC-I Qa- 1^*b*^ have indeed been associated with enhanced viral control against mousepox infections (Fang et al. 2011). Qa- 1^*b*^ is normally loaded with small peptides derived from other self MHC-I molecules, which forms the natural ligand for the inhibiting CD94/NKG2A receptor. After mouse pox infection, these peptide-MHC complexes are no longer recognized by inhibiting receptors but they bind to activating receptors. The molecular mechanisms underlying this “altered-self” detection remain unknown, as it is not clear whether it is a viral or a newly expressed self peptide that is engaging the activating receptor. Nevertheless, this observation clearly highlights the importance of activating NK cell receptors in recognizing peptide-MHC complexes during viral infections, calling for further studies.

Our recent analysis of the peptides presented by HLA molecules before and after infection with measles virus (MV) has shed light on the required specificity for an inhibiting KIR to detect altered-self (Carrillo-Bustamante and et al. 2015a). To be able to detect the changes in the peptide pool induced after a viral infection, inhibiting KIRs need to be sufficiently specific, i.e., they must be able to discriminate between any unique amino acid pairs or groups of amino acid groups in P7 and P8 (unpublished results). Because of this required specificity, an individual would need more than one inhibiting KIR to detect changes in the peptide repertoire after a viral infection, indicating that a diverse KIR repertoire is advantageous to successfully detect altered self. Further experimental elucidation of the KIR motifs in the MHC presented peptides is necessary to validate these predictions.

## Concluding remarks

The evolution of NK cell receptors in different species led to a fascinating complexity. Most NK cell receptors interact with the highly polymorphic MHC molecules, resulting in receptor-ligand pairs that individualize the immune system, a process that is thought to improve hosts’ survival. Yet, the exact evolutionary advantage of these expanded genotypes has remained unresolved. In this review, we have discussed several hypothesis that possibly underly the diversification of the NK cell receptor complex and provided some mechanistic insights into these viral-driven hypotheses, i.e., viral evolution of decoys, peptide sensitivity, and selective MHC-downregulation.

In the last 5 years, we have studied each of the hypotheses independently using computational and mathematical modeling. These studies revealed that each mechanism revised here can contribute, albeit in different degrees, to the evolution of polygenicity and polymorphism in the clusters encoding NK cell receptors. The studies reviewed here mirror the complexity of the biological process, as we now have several explanations for the complex biological question why NK cell receptors are specific, polygenic, and polymorphic. All these processes are most likely intertwined, simultaneously exerting pressures on hosts to evolve the functional NKR-MHC pairs which render NK cells protective. Therefore, the development of a more general model incorporating all three mechanisms (i.e., viral evolution of decoys, peptide sensitivity, and selective MHC-downregulation) is essential to quantify their contribution to the required genetic diversity for a population’s long-term survival. A more complete model might shed also light onto how different pathogen interactions (i.e., several evasion mechanisms) influence the fixation of alternative NK receptor systems in different species. The evolutionary contribution of a diverse NK cell receptor repertoire to reproductive success remains still open and also calls for another extension of our models.

Importantly, the degree of genetic diversity that evolves in our simulations depends strongly on how the host’s protection is modeled. In all our studies, the existence of MHC-I decoys (Carrillo-Bustamante et al. 2013, 2014, 2015c) results in the evolution inhibiting receptors with a high degree of specificity recognizing very few MHC molecules in the population. This high specificity that hosts required to clear decoy-encoding viruses (which in turn exerts a stronger selection pressure on the NK cell receptors) depends on whether *at least one* or *all* inhibiting receptors in the licensed repertoire should be protective. Based on the experiments of MCMV infected 129/J mice which showed that one interaction between the inhibiting Ly49I and the decoy protein m157 is sufficient for the host to succumb the infection (Smith et al. 2002), we assumed that *all* inhibiting receptors must be protective to clear the infection. However, it is counterintuitive to have one inhibiting interaction dominating the host’s NK cell response. Theoretically, if one inhibiting receptor is able to detect missing-self (i.e., to be protective), the NK cell subsets carrying that receptor should proliferate and provide some degree of protection. Indeed, relaxing this assumption (i.e., that a host needs *at least one* protective inhibiting receptors to clear the infection) results in a lower degree of diversity as shown by our model of selective downregulation (Carrillo-Bustamante et al. 2015b). The actual protection probably lies between *all* and *at least one* protective inhibiting NK cell receptors, but the current understanding of the contribution of each NK cell subset to host’s protection during a viral infection remains limited.

Our computational models provided insightful mechanistic insights into viral selection pressures driving the evolution of NK cell receptors. For future extensions of these models, determining the complex, still unresolved, molecular and cellular processes of NK cells is crucial. Shedding light on these mechanisms, including the precise role of MHC-I molecules in NK cell maturation, the exact mechanisms of NKR repertoire acquisition, and the expansion of different NK cell subsets during viral infections, will allow us to better study the effects of NK cell education, and the diversity of NK cell subsets in fighting pathogens and survival.
